# Neonatal sepsis definitions for randomised–controlled trials: challenges and approaches

**DOI:** 10.1093/ije/dyag154

**Published:** 2026-08-18

**Authors:** Chiara Minotti, Louise F Hill, Michelle N Clements, Wolfgang Stohr, Ann Sarah Walker, Julia A Bielicki

**Affiliations:** Paediatric Research Centre, University Children’s Hospital Basel (UKBB), Basel, Switzerland; Department of Clinical Research (DKF), Faculty of Medicine, University of Basel, Basel, Switzerland; Centre for Neonatal and Paediatric Infection (CNPI), Institute for Infection & Immunity, City St. George’s University of London, London, United Kingdom; Centre for Neonatal and Paediatric Infection (CNPI), Institute for Infection & Immunity, City St. George’s University of London, London, United Kingdom; Medical Research Council (MRC) Clinical Trials Unit, University College London, London, United Kingdom; Medical Research Council (MRC) Clinical Trials Unit, University College London, London, United Kingdom; Medical Research Council (MRC) Clinical Trials Unit, University College London, London, United Kingdom; Paediatric Research Centre, University Children’s Hospital Basel (UKBB), Basel, Switzerland; Department of Clinical Research (DKF), Faculty of Medicine, University of Basel, Basel, Switzerland; Centre for Neonatal and Paediatric Infection (CNPI), Institute for Infection & Immunity, City St. George’s University of London, London, United Kingdom

## Defining neonatal sepsis for trials: a major challenge

Neonatal infections lead to >550 000 deaths worldwide every year; furthermore, 6.9 million episodes of possible serious bacterial infections are estimated to occur in infants aged between 0 and 59 days in low- and middle-income countries (LMICs) annually [1]. In contrast to adult and paediatric sepsis, for which periodically updated consensus frameworks exist (most recently Sepsis-3 and Phoenix [2–5]) and provide a shared conceptual anchor for both clinical use and trial design, the absence of an analogous, widely adopted standard for neonates underscores why definitional variability remains so pronounced in this age group [6–9].

Neonatal sepsis is difficult to diagnose definitively, as the symptoms are notably unspecific and often overlap with those of other neonatal pathologies [10]. Furthermore, presentation and risk factors vary between preterm, term, and comorbid infants, with those affected displaying relatively limited signs and symptoms or not appearing to be critically ill [11–13]. Causative organisms often remain unidentified; among infants evaluated for suspected sepsis, blood culture positivity is typically low (∼15%; e.g. the NeoOBS study reported 18% blood culture positivity), although this does not directly indicate the proportion of true sepsis episodes [14, 15].

Researchers face the parallel challenge of defining trial eligibility in ways that are both scientifically rigorous and clinically meaningful. For example, clinically, sepsis is dynamic and evolves over time, whereas clinical trials require the assessment of eligibility at a fixed, operationally defined time point.

The result is a patchwork of definitions that diverge between settings and institutions, and even between trials within the same field. This variability poses a major obstacle to progress: it undermines trial interpretability, hampers comparability across studies, and risks producing results that are difficult to translate into clinical decision-making [6, 11].

## Published neonatal sepsis definitions

Attempts to define sepsis over the past 25 years have produced a myriad of criteria, but none has achieved universal acceptance for neonatal sepsis (Table 1) [16]. Clinically, the overriding priority is sensitivity: definitions are designed to capture all possible cases, even if this means treating many infants who ultimately do not have sepsis. This ‘sensitivity-first’ approach is clinically justifiable, but also carries costs: large numbers of uninfected infants receive empiric antibiotics, contributing to antimicrobial resistance, disruption of the developing microbiome, and additional strain on health systems. These broader consequences reinforce the need for clearer justification when adapting clinical definitions for use in trials. Balancing safety with specificity is therefore not only a methodological problem, but also a stewardship issue.

**Table 1:** Summary of sepsis definitions and scores over time across adults, children, and neonates.

Consensus	Year	Definition	Age group	Sepsis criteria	Severe sepsis criteria
Sepsis-1, Bone *et al*. [2]	1992	Overwhelming inflammatory response to infection, as evidenced by SIRS	Adult sepsis	Infection plus two SIRS criteria^a^	Sepsis associated with organ dysfunction, hypoperfusion, or hypotension
Sepsis-2, Levy *et al*. [3]	2003	Overwhelming inflammatory response to infection, including an expanded list of potential signs and symptoms of sepsis, reflecting bedside clinical experience	Adult sepsis	Infection plus two or more signs or symptoms of sepsis, including SIRS criteria, inflammatory markers (i.e. increased CRP or procalcitonin), haemodynamic, organ-dysfunction, and tissue-perfusion markers	Sepsis complicated by organ dysfunction
IPSCC, Goldstein *et al*. [16]	2005	SIRS in the presence of suspicion or definite infection in infants aged >37 weeks	Neonatal and paediatric sepsis Newborn: 0 days–1 week Neonate: 1 week–1 month Premature infants not included	Infection plus two SIRS criteria resulting from infection; among SIRS criteria^b^ at least one must be abnormal temperature or WBC	Sepsis with cardiovascular organ dysfunction, ARDS, or dysfunction of two or more organs
EMA, Expert Meeting on Neonatal and Paediatric Sepsis [17]	2010	At least two clinical signs and two laboratory signs together with suspected or proven infection (positive culture, microscopy, or PCR)	Neonatal and paediatric sepsis Neonates: birth to <1 month	Suspected/proven infection with at least two clinical signs and two laboratory signs^c^	Severe sepsis is not a separate entity
Sepsis-3, Singer *et al*. [4]	2016	Life-threatening organ dysfunction caused by a dysregulated host response to infection (SIRS no longer included in the definition of sepsis)	Adult sepsis	Infection plus infection-related acute organ dysfunction Life-threatening acute organ dysfunction: increase from baseline of ≥2 points in Sequential Organ Failure Assessment score	Severe sepsis is no longer a separate entity
WHO IMCI clinical algorithm [19]	2019	Possible serious bacterial infection in young infants	Neonatal and paediatric sepsis Young infants: >1 week Newborns (<1 week) not included	Neurologic and respiratory signs and symptoms General signs and symptoms suggestive of infection^d^	Not applicable
nSOFA score, Wynn *et al*. [21]	2020	Life-threatening organ dysfunction caused by a dysregulated host response to infection Late-onset sepsis—‘all of the following: (1) the event occurred after the 3rd day of life, (2) subjective clinician concern for serious infection (demonstrated by a blood culture being performed, empiric antimicrobial treatment started at evaluation and continued for at least 7 days or until death), and (3) a single bacterial/fungal pathogen isolated from blood or death with necrotizing enterocolitis’	Neonatal sepsis: operational definition of organ dysfunction applicable specifically to neonates that predicts mortality in the setting of presumed infection	(i) Respiratory score (ii) Cardiovascular score (iii) Haematologic score^e^	Not applicable
NeoSep Severity Score, NeoSep1 [22]	2022	Clinical signs in the 24 h preceding start of episode Moderate to high risk of death from current sepsis episode, based on NeoSep Severity Score (adapted from WHO pSBI criteria for hospitalized neonates with sepsis and based on the data generated from the NeoOBS study) IV antibiotics about to be started OR not received >24 h before episode onset	Neonatal sepsis	Time in hospital Gestational age Birthweight Congenital anomalies Abnormal temperature Maximum respiratory support Abdominal distension Difficulty in feeding Evidence of shock Lethargy/no or reduced movement^f^	Not applicable
Phoenix score, Schlapbach *et al*. [5]	2024	Dysregulated host response to infection, resulting in acute organ dysfunction Sepsis: suspected infection and Phoenix Sepsis Score of ≥2 points Septic shock: Sepsis with ≥1 cardiovascular point(s)	Neonatal and paediatric sepsis (preterm infants with post-conceptional age of <37 weeks excluded)	Life-threatening organ dysfunction with suspected or confirmed infection Organ dysfunction may include respiratory, cardiovascular, coagulation, and neurologic dysfunction^g^	Severe sepsis is no longer a separate entity

Based on Meyer *et al*. [16].

a SIRS criteria: temperature >38°C or <36°C; heart rate >90 beats/min; respiratory rate >20 breaths/min or PaCO2 < 32 mmHg; or WBC >12 000/mm^3^ or <4000/mm^3^ or >10% immature bands.

b SIRS criteria: heart rate >180 beats/min or <100 beats/min; respiratory rate >50 breaths/min (0 days–1 week)/>40 breaths/min (1 week–1 month); WBC >34 000/mm^3^ (0 days–1 week)/>19 500 or <5000/mm^3^ (1 week–1 month) or >10% immature neutrophils; systolic blood pressure <65mmHg (0 days–1 week) or <75mmHg (1 week–1 month).

c Clinical and laboratory signs: body temperature abnormality; bradycardia, tachycardia, or rhythm instability; oliguria; hypotension; mottled skin; impaired peripheral perfusion; petechial rash; scleraema; apnoea; tachypnoea; increased oxygen requirements; requirements for ventilation support; feeding intolerance; poor sucking; abdominal distention; irritability; lethargy; hypotonia; WBC count abnormality; immature-to-total (I/T) > 0.20; thrombocytopaenia; CRP >15 mg/L; glucose intolerance; base excess less than −10 mEq/L; serum lactate >2 mmol/L.

d Convulsions or Fast breathing (60 breaths per minute or more) or Severe chest indrawing or Nasal flaring or Grunting or Bulging fontanelle or Pus draining from ear or Umbilical redness extending to the skin or Fever (≥37.5°C or feels hot) or low body temperature (<35.5°C or feels cold)—axillary temperature or Many or severe skin pustules or Lethargic or unconscious or Less than normal movement.

e (i) Respiratory score: the need for mechanical ventilation and oxygen requirement (score range 0–8); (ii) cardiovascular score: the need for inotropic support including the use of corticosteroid support (for presumed adrenal insufficiency or catecholamine-resistant shock) (score range 0–4); (iii) haematologic score: the presence and degree of thrombocytopenia (score range 0–3).

f Time in hospital: ≤10 days (score value 1); Gestational age: <37 weeks (score value 1); Birthweight: >2 kg/1–2 kg/<1 kg (score range 0–2); Congenital anomalies (score value 2); Temperature: <35.5°C/35.5–37.9°C/38–38.9°C/≥39°C (score range 1/0–2); Maximum respiratory support: none/oxygen supplementation/continuous positive airway pressure (CPAP), bilevel positive airway pressure (BiPAP), high-flow nasal cannula (HFNC)/invasive ventilation (score values 0/2/3/3); Abdominal distension (score value 1); Difficulty in feeding (score value 1); Evidence of shock including cold peripheries (score value 1); Lethargy only (score value 1); No movement or movement only on stimulation ± lethargy (score value 2).

g (i) Respiratory: PaO2: FIO2 ratio or SpO2: FIO2 ratio, any respiratory support, invasive mechanical ventilation (0–3 points); (ii) cardiovascular: no. of vasoactive medications, lactate, mean arterial pressure by age (0–6 points); (iii) coagulation: platelet count, International Normalized Ratio (INR), D-dimer, fibrinogen (0–2 points); (iv) neurological: Glasgow Coma Scale, pupil reactivity (0–2 points).

SIRS, systemic inflammatory response syndrome; bpm or beats/min, beats per minute; WBC, white blood cell; CRP, C-reactive protein; IPSCC, International Paediatric Sepsis Consensus Conference; ARDS, acute respiratory distress syndrome; EMA, European Medicines Agency; PCR, polymerase chain reaction; WHO, World Health Organization; IMCI, Integrated Management of Childhood Illness; nSOFA, neonatal Sequential Organ Failure Assessment; pSBI, possible serious bacterial infection.

Several consensus definitions illustrate these difficulties. The 2005 International Paediatric Sepsis Consensus Conference extended the systemic inflammatory response syndrome (SIRS) framework to neonates, but its reliance on white cell counts and temperature was poorly suited to newborn physiology [17]. The 2010 European Medicines Agency criteria require a combination of clinical and laboratory signs plus suspected or proven infection, but rely on laboratory tests that have variable availability, particularly in LMICs [18, 19]. By contrast, the 2019 World Health Organization (WHO) Integrated Management of Childhood Illness (IMCI) clinical algorithm, designed with resource-limited settings in mind, relies on clinical signs alone to guide antibiotic initiation [20, 21].

More recently, attention has shifted to organ-dysfunction scores. The neonatal Sequential Organ Failure Assessment (nSOFA) score operationalises dysfunction across respiratory, cardiovascular, and haematologic domains. nSOFA predicts mortality amongst infected neonates, but depends on advanced monitoring and treatments rarely available outside high-resource settings [22]. The Phoenix criteria, developed by using data from both high- and low-income contexts, also incorporates organ dysfunction while attempting to reduce the reliance on extensive laboratory testing, though validation excluded preterm infants (born at <37 weeks’ gestational age) and still requires access to some investigations not universally available [5, 23]. Finally, pragmatic approaches that focus largely on outlining trial eligibility, such as the NeoSep Severity Score, have focused on clinical signs associated with mortality risk, aiming to balance generalisability and applicability across diverse trial contexts [15, 24].

Despite these advances, all published definitions have limitations for use as a universal randomised controlled trial (RCT) eligibility criterion. They generally rely on metrics and monitoring not available in much of the world (e.g. nSOFA), are misaligned with neonatal physiology (e.g. SIRS), are overly sensitive for trial enrolment (e.g. WHO IMCI), exclude relevant neonatal subpopulations (e.g. Phoenix), or remain disconnected from the real-time bedside decisions that clinicians must make. Crucially, organ-dysfunction scores predict mortality, but do not themselves define sepsis, and case definitions are often limited to arbitrary subgroups (e.g. early vs. late-onset, suspected vs. proven sepsis) that reduce generalisability. In practice, neonatal sepsis definitions remain fragmented, resource-dependent, and poorly harmonised across clinical and research domains.

## Why definitions vary: clinical and research drivers

Variability in neonatal sepsis definitions reflects real-world pressures as well as research shortcomings. As above, the foremost clinical priority is sensitivity: missing a true case of sepsis can be catastrophic, so definitions aimed at clinical applications err towards including borderline or uncertain cases. This means that many infants start antibiotics on suspicion alone, with false positives accepted as the lesser risk. Globally, definitions are shaped by resource availability. In high-income settings, laboratory markers and microbiological confirmation are often expected and routinely available, while, in LMICs, syndromic bedside algorithms based solely on clinical signs remain the pragmatic choice.

Therefore, two factors help explain to why eligibility definitions vary so widely across neonatal sepsis trials. First, the clinical impact of missing a true case is extremely high, particularly in very preterm infants, who deteriorate rapidly with little opportunity for subsequent intervention. As a result, even in studies, researchers commonly adopt highly sensitive criteria that minimise false negatives at the cost of including many infants who ultimately do not have bacterial sepsis. Second, a substantial proportion of infants fall into the broad category of ‘probable’ or culture-negative sepsis, for which the true underlying diagnoses remain uncertain, ranging from viral infections to non-infectious inflammatory conditions. This diagnostic ambiguity inevitably shapes trial definitions, as investigators differ in how much of this heterogeneous group they include within their target population.

For RCTs, however, one key problem is that eligibility criteria are often not explicitly justified in relation to a ‘population intervention control outcome’ (PICO) framework—i.e. they do not clearly specify the patient population to whom results will apply, the intervention being tested, or the clinical decision that the trial is meant to inform. Instead, neonatal sepsis definitions may be adopted from clinical practice or constructed pragmatically, but without an explicit rationale for why they fit the trial question. The consequence is wide heterogeneity in trial populations and a weak link between trial outcomes and the clinical scenarios in which their findings are meant to guide practice.

Another source of variability for trials arises from the way in which they are positioned along the efficacy–effectiveness spectrum. Efficacy trials, designed to isolate biological effects under ideal conditions, often use highly selective eligibility criteria that may not reflect clinical reality. By contrast, effectiveness trials aim to test interventions in the populations and settings in which they will actually be used, requiring broader and more pragmatic definitions. In trials investigating antibiotic treatment, efficacy is often already established in adults and dosing studies enable the use of antibiotics at efficacious doses; most trials beyond Phase 2 should logically focus on effectiveness. This means that eligibility definitions should mirror the clinical populations in whom the intervention, such as a new antibiotic regimen, would realistically be deployed, rather than relying on narrow or artificial criteria. Balancing this, although sometimes argued for generalisability, including large numbers of neonates who actually do not have sepsis, and will therefore do equally well (or badly) regardless of the intervention, leads to dilution bias.

Trial-design choices between superiority and non-inferiority hypotheses also influence definitions. Superiority trials can tolerate more inclusive eligibility, as including some infants without sepsis dilutes the treatment effect, but does not fundamentally undermine interpretability. In contrast, non-inferiority trials are highly vulnerable to such dilution bias: enrolling infants without sepsis (and therefore not expected to benefit from the trialled intervention) risks masking real differences between treatments. For such designs, definitions need to be more specific, targeting infants in whom both the intervention and comparator could plausibly alter outcomes.

## Evidence from RCTs: heterogeneous and poorly linked definitions

We reviewed neonatal sepsis definitions (focusing on those adopted for eligibility and not outcome assessment) used in trials from a recently published systematic review [25] and updated by searching PubMed and ClinicalTrials.gov without date or language restrictions (details provided in Supplementary Materials).

Our review of 29 neonatal sepsis RCTs illustrates how heterogeneous definitions have become. Across trials, eligibility criteria combined varying elements of clinical signs, laboratory markers, microbiological results, and maternal or neonatal risk factors, with no consistent framework (Supplementary Tables 1 and 2). Some trials relied on culture-confirmed sepsis, achieving high specificity, but at the cost of excluding most sepsis cases, given the low culture-positivity rates in neonates. The focus on microbiological results diverges from the most recently established sepsis scores, which have instead shifted towards pathophysiological aspects and organ dysfunction. Other trials enrolled infants on the basis of broad suspected sepsis criteria, maximising sensitivity but including many infants whose outcomes could not plausibly be altered by the intervention. This heterogeneity extended beyond the choice of diagnostic domains to how criteria were operationalised. For example, two studies required two positive cultures for coagulase-negative staphylococci while others excluded these organisms altogether [26, 27]. The choice of laboratory markers also varied, often with inconsistent thresholds. Relatively few trials explicitly referenced existing sepsis definitions or validated scores and, when they did, application was often partial. For example, there were trials testing the same intervention that used different eligibility criteria. This was the case for Mehta *et al*. [28] and Banupriya *et al*. [29], both of which were set in LMICs and investigated oral zinc supplementation to reduce mortality in suspected neonatal sepsis. The former applied International Classification of Diseases and WHO classifications and included infants with clinically probable sepsis and at least one amongst either laboratory confirmation [‘positive sepsis screen’ with suggestive C-reactive protein (CRP), white blood cell count, immature-to-total neutrophil ratio], maternal risk factors, or radiological evidence of pneumonia, whereas the latter trial required clinical signs and at least two positive laboratory tests from CRP, micro erythrocyte sedimentation rate (microESR), and band cell count, without referencing an existing definition. Another example is provided by non-inferiority trials of antibiotic duration that used neonatal sepsis definitions with varying specificity, including study populations with possibly different baseline risks. Dutta *et al*. included infants with suspected sepsis for which a blood culture was sent and for which the treating physician decided to start antibiotics [30], whereas Rohatgi *et al*. restricted enrolment to infants with clinical and laboratory signs of sepsis confirmed with a positive blood culture [31]. These examples illustrate that heterogeneity is not limited to isolated criteria, but affects the clinical phenotype being enrolled, baseline risk, and therefore the interpretation and comparability of treatment effects.

The result is a set of trials that are difficult to interpret collectively. Meta-analysis is hampered when eligibility definitions differ not only in sensitivity and specificity, but also in their underlying rationale. Equally importantly, the rationale connecting eligibility definitions to the decision-making context was often not made explicit. Few justified why their criteria matched the intervention studied, the setting in which it would be used, or the patient population to whom results should apply. In practice, this means that, while many RCTs generate internally valid results, their external validity is often unclear. In short, neonatal sepsis RCTs to date illustrate the consequences of definitional drift: heterogeneity in inclusion criteria that undermines comparability, weakens evidence synthesis, and obscures the link between trial findings and real-world clinical management.

## Consequences: risk of limited utility of current trials

The immediate consequence of definitional heterogeneity is that neonatal sepsis trials cannot easily be compared or combined. Meta-analysis depends on commensurable study populations, yet the wide variation in eligibility criteria means that aggregated results often mix fundamentally different patient groups. This dilutes effect estimates and obscures meaningful conclusions.

Equally importantly, unclear or poorly justified eligibility definitions limit the clinical utility of trial results. When criteria are not explicitly linked to the intervention under study or the management decision being tested, such as antibiotic choice, treatment duration, or the escalation of care, clinicians cannot easily judge whether findings apply to their own patients. As a result, trials risk producing evidence that is internally valid, but externally unhelpful.

Finally, the absence of a single universal definition does not mean that ‘anything goes’. On the contrary, the lack of harmonisation makes it even more important that trialists clearly justify their chosen definition, explain how it relates to PICO, and show how it aligns with clinical practice in the setting in which the intervention will be used. Without this, neonatal sepsis RCTs risk continuing to generate evidence that is fragmented, difficult to synthesize, and of limited relevance to the populations most affected by the disease.

## A way forward: adaptable, transparent, and pragmatic definitions

**Figure 1:**
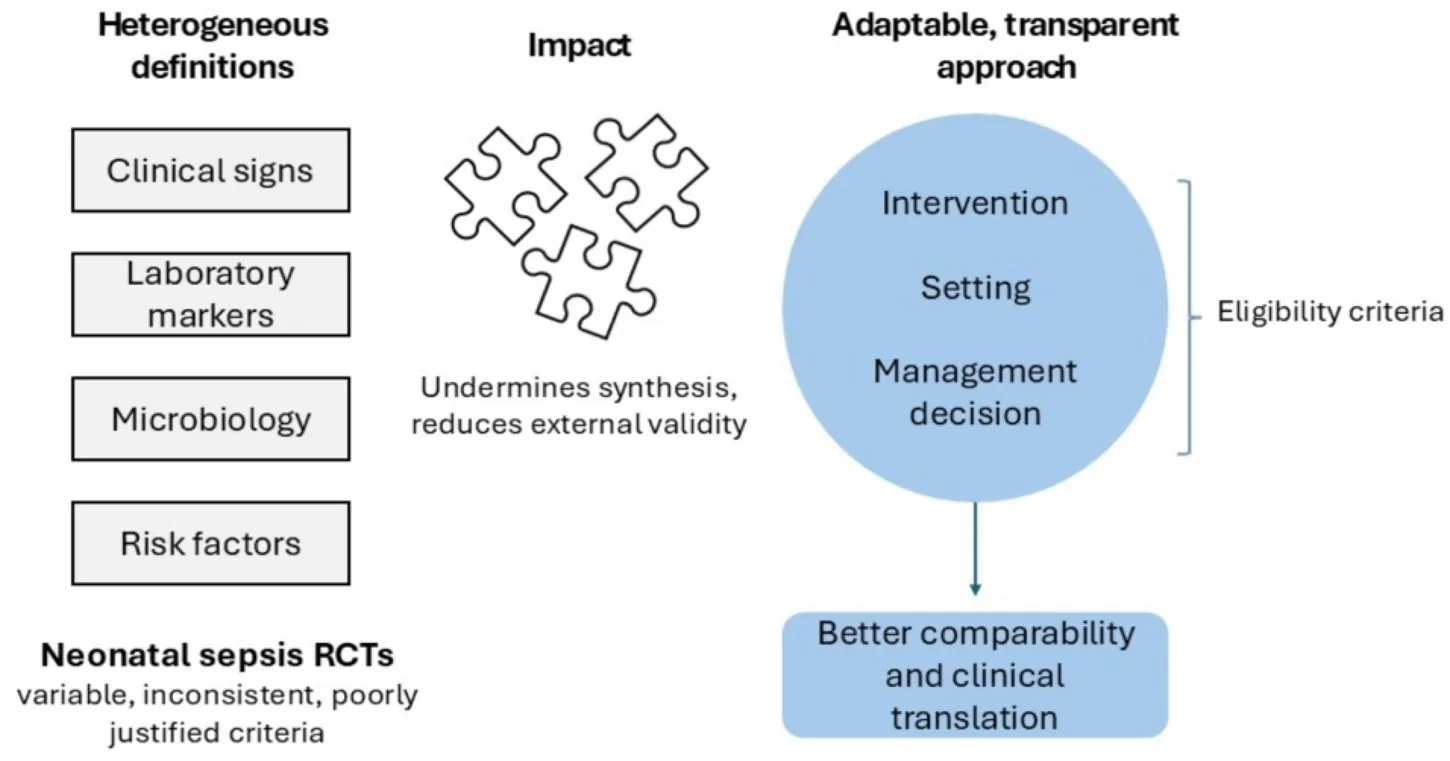
Defining neonatal sepsis for randomised controlled trials (RCTs).

The limitations of existing neonatal sepsis definitions do not point towards the need for a single universal standard. Given the diversity of settings, pathogens, and interventions, such a definition would be neither feasible nor desirable. Instead, what is required is an adaptable approach that recognises the variability of clinical practice, but insists on transparency and justification in trial design (Fig. 1). Applying a PICO-derived framework explicitly helps clarify what degree of specificity or sensitivity is appropriate. For instance, a superiority trial testing the value of a diagnostic biomarker should prioritise specificity to avoid false positives; a trial in LMIC settings must rely on universally available clinical signs; and a non-inferiority trial of antibiotic duration requires high specificity (e.g. culture-confirmed sepsis) to avoid dilution bias, arguing that generalisability of results to infants who do not actually need antibiotics at all can be assumed. Such decisions cannot be universal, but should be transparently justified.

At a minimum, RCTs should make explicit the target population to whom results are intended to apply and how their definitions ensure generalizability to this population; the intervention and management decisions that the trial is meant to inform; the setting and diagnostic resources assumed at screening; the rationale for including or excluding major diagnostic domains; and the likely consequences of misclassification for the chosen trial design (Table 2).

**Table 2:** Proposed minimum justifications for operationalisation of neonatal sepsis to define trial population.

Item number	Justification	Example	Illustrative example from NeoSep1 protocol (ISRCTN48721236)
1	Target population: State clearly which infants the results are intended to apply to	‘Hospitalised neonates with suspected late-onset sepsis receiving IV antibiotics’ rather than simply ‘neonatal sepsis’	Hospitalised neonates aged ≤28 days (postnatal age) and weighing ≥1000 g with two or more specific clinical signs of sepsis, a NeoSep Severity Score [15] of ≥5, and IV antibiotic treatment planned or initiated <24 h previously; clear additional exclusion criteria
2	Intended setting and resource assumptions: state whether the definition is designed for district hospitals, tertiary NICUs, LMIC settings, or high-resource settings, and which investigations are assumed available at screening	‘Eligibility based on clinical signs only, as CRP and continuous monitoring are not routinely available in the district hospitals included in this trial’	LMIC tertiary hospitals; inclusion criteria are based only on clinical factors. Baseline FBC, CRP U&Es, and LFTs are required within 72 h of randomisation, but are not part of eligibility criteria
3	Timing of eligibility assessment: define when and over what window sepsis is assessed for enrolment	‘Eligibility assessed at antibiotic initiation; screening laboratory values must be available within 48 hours before randomisation’	Enrols infants with clinical signs of sepsis prior to initiation of IV antibiotics or within 24 h of starting antibiotics for the current sepsis episode at randomisation
4	Diagnostic domains included and excluded: specify which clinical, laboratory, microbiological, and risk-factor domains are used, and why	‘Clinical signs plus CRP used; maternal risk factors recorded but not required for enrolment’	Enrols infants based on clinical signs of sepsis only; laboratory testing and blood culture are incorporated into screening and follow-up (blood culture and CRP). Infants meeting the inclusion criteria are still eligible even if laboratory results and blood culture (done for baseline assessment) are pending. Culture confirmation is not required for entry, but microbiology remains relevant to subsequent management
5	Expected impact of misclassification on trial design: explain whether the design prioritises sensitivity or specificity and why	‘Because this is a non-inferiority trial, specificity was prioritised to reduce dilution bias from infants unlikely to benefit. We assume that if the intervention is non-inferior to control in the population of infants who require antibiotics, this will generalise to infants without sepsis who nevertheless receive antibiotics’	By including only neonates with at least two clinical signs of sepsis and a NeoSep Severity Score of ≥5, the trial focuses on newborns with clinical signs of sepsis at high risk of mortality, who would universally receive antibiotics: demonstrating superiority in this population would generalize to neonates at lower risk
6	Relationship to existing definitions or scores: map the trial definition to WHO IMCI, EMA, nSOFA, NeoSep, Phoenix, or another framework where relevant	‘Eligibility criteria were informed by WHO IMCI signs but adapted to hospital-based screening’	Clinical signs based on the WHO pSBI classification and validated within the NeoOBS study. Anchors response assessment to NeoOBS-derived tools, including the NeoSep Severity Score, and cites NeoOBS as informing trial design

NICU, neonatal intensive care unit; LMIC, low- and middle-income countries; CoNS, coagulase-negative staphylococci; WHO, World Health Organization; IMCI, Integrated Management of Childhood Illness; pSBI, possible serious bacterial infection; nSOFA, neonatal Sequential Organ Failure Assessment; EMA, European Medicines Agency; IV, intravenous; CRP, C-reactive protein; FBC, full blood count; U&E, urea and electrolytes; LFTs, liver function tests.

Harmonisation, then, should be about process rather than prescription: not the imposition of a single rigid definition, but the consistent expectation that trial definitions are transparent, justified, and pragmatically aligned with both the intervention and the setting. Such an approach would strengthen the external validity of individual trials, improve the comparability of results across studies, and ultimately ensure that research evidence better serves clinical decision-making in neonatal sepsis.

## Conclusion

Neonatal sepsis definitions remain highly variable across both clinical practice and research, including RCTs. This variability reflects varying imperatives for sensitivity, global differences in access to diagnostics and therapeutics, and a lack of clarity in how trial definitions are explicitly justified in relation to the question and design of the trial. Our review shows that neonatal sepsis RCTs have used heterogeneous and inconsistently justified definitions, combining clinical signs, laboratory markers, microbiological results, and risk factors without explicitly linking them to the clinical decisions that their findings are meant to inform. As a result, trials are difficult to compare, synthesis is undermined, and external validity is often limited.

A key distinction is between eligibility definitions, which determine which infants receive the intervention under study, and outcome definitions, which determine whether the intervention was effective. Eligibility definitions are closely tied to the underlying clinical condition that prompts treatment and therefore require particular clarity and justification. Outcome definitions, by contrast, may appropriately allow greater flexibility and may incorporate a broader range of clinical, microbiological, or composite measures, depending on the research question.

A universal definition is unlikely to be desirable. Instead, we argue for an adaptable, transparent, and pragmatic approach in which trialists justify their eligibility criteria in relation to intervention, setting, and management decision. This shift would not only enhance the comparability and utility of neonatal sepsis trials, but also ensure that research evidence translates more effectively into clinical care for the newborns who need it most.

## Ethics approval

Ethical approval was not required, as this is an opinion piece based on a review of the literature.

## Supplementary Material

dyag154_Supplementary_Data

## Data Availability

The data underlying this article are either available in the article and its online supplementary material or will be shared upon reasonable request to the corresponding author.

## References

[1] WHO. *Newborn Health*. https://www.who.int/health-topics/newborn-health (14 April 2026, date last accessed).

[2] Bone RC , Balk RA, Cerra FB et al Definitions for sepsis and organ failure and guidelines for the use of innovative therapies in sepsis. The ACCP/SCCM Consensus Conference Committee. American College of Chest Physicians/Society of Critical Care Medicine. Chest 1992;101:1644–55. 10.1378/chest.101.6.1644 1303622

[3] Levy MM , Fink MP, Marshall JC et al; International Sepsis Definitions Conference. 2001 SCCM/ESICM/ACCP/ATS/SIS International Sepsis Definitions Conference. Intensive Care Med 2003;29:530–8. 10.1007/s00134-003-1662-x 12664219

[4] Singer M , Deutschman CS, Seymour CW et al The Third International Consensus Definitions for Sepsis and Septic Shock (Sepsis-3). Jama 2016;315:801–10. 10.1001/jama.2016.0287 PMC4968574 26903338

[5] Schlapbach LJ , Watson RS, Sorce LR et al; Society of Critical Care Medicine Pediatric Sepsis Definition Task Force. International consensus criteria for pediatric sepsis and septic shock. Jama 2024;331:665–74. 10.1001/jama.2024.0179

[6] Wynn JL. Defining neonatal sepsis. Curr Opin Pediatr 2016;28:135–40. 10.1097/mop.0000000000000315 PMC4786443 26766602

[7] McGovern M , Giannoni E, Kuester H et al; Infection, Inflammation, Immunology and Immunisation (I4) section of the ESPR. Challenges in developing a consensus definition of neonatal sepsis. Pediatr Res 2020;88:14–26. 10.1038/s41390-020-0785-x 32126571

[8] Menon K , Schlapbach LJ, Akech S et al; Pediatric Sepsis Definition Taskforce of the Society of Critical Care Medicine. Criteria for pediatric sepsis-a systematic review and meta-analysis by the pediatric sepsis definition taskforce. Crit Care Med 2022;50:21–36. 10.1097/ccm.0000000000005294 PMC8670345 34612847

[9] Strunk T , Molloy EJ, Mishra A, Bhutta ZA. Neonatal bacterial sepsis. Lancet 2024;404:277–93. 10.1016/s0140-6736(24)00495-1 38944044

[10] Seale AC , Blencowe H, Manu AA et al; pSBI Investigator Group. Estimates of possible severe bacterial infection in neonates in sub-Saharan Africa, south Asia, and Latin America for 2012: a systematic review and meta-analysis. Lancet Infect Dis 2014;14:731–41. 10.1016/s1473-3099(14)70804-7 PMC4123782 24974250

[11] Molloy EJ , Wynn JL, Bliss J et al; on behalf of the Infection, Inflammation, Immunology and Immunisation (I4) section of the ESPR. Neonatal sepsis: need for consensus definition, collaboration and core outcomes. Pediatr Res 2020;88:2–4. 10.1038/s41390-020-0850-5 32193517

[12] Verstraete E , Boelens J, De Coen K et al Healthcare-associated bloodstream infections in a neonatal intensive care unit over a 20-year period (1992-2011): trends in incidence, pathogens, and mortality. Infect Control Hosp Epidemiol 2014;35:511–8. 10.1086/675836 24709719

[13] Cotten CM. Adverse consequences of neonatal antibiotic exposure. Curr Opin Pediatr 2016;28:141–9. 10.1097/mop.0000000000000338 PMC4845665 26886785

[14] Klingenberg C , Kornelisse RF, Buonocore G et al Culture-negative early-onset neonatal sepsis - at the crossroad between efficient sepsis care and antimicrobial stewardship. Front Pediatr 2018;6:285. 10.3389/fped.2018.00285 PMC6189301 30356671

[15] Russell NJ , Stöhr W, Plakkal N et al Patterns of antibiotic use, pathogens, and prediction of mortality in hospitalized neonates and young infants with sepsis: a global neonatal sepsis observational cohort study (NeoOBS). PLoS Med 2023;20:e1004179. 10.1371/journal.pmed.1004179 PMC10249878 37289666

[16] Meyer NJ , Prescott HC. Sepsis and septic shock. N Engl J Med 2024;391:2133–46. 10.1056/NEJMra2403213 39774315

[17] Goldstein B , Giroir B, Randolph A, International Consensus Conference on Pediatric Sepsis. International pediatric sepsis consensus conference: definitions for sepsis and organ dysfunction in pediatrics. Pediatr Crit Care Med 2005;6:2–8. 10.1097/01.Pcc.0000149131.72248.E6 15636651

[18] EMA. *Report on the Expert Meeting on Neonatal and Paediatric Sepsis London*. *2010*. https://www.ema.europa.eu/en/documents/report/report-expert-meeting-neonatal-and-paediatric-sepsis_en.pdf (14 April 2026, date last accessed).

[19] Tuzun F , Ozkan H, Cetinkaya M et al Is European Medicines Agency (EMA) sepsis criteria accurate for neonatal sepsis diagnosis or do we need new criteria? PLoS One 2019;14:e0218002. 10.1371/journal.pone.0218002 PMC6553766 31170237

[20] WHO. *Integrated management of childhood illness*. https://www.who.int/teams/maternal-newborn-child-adolescent-health-and-ageing/child-health/integrated-management-of-childhood-illness (14 April 2026, date last accessed).

[21] Carai S , Kuttumuratova A, Boderscova L et al Review of Integrated Management of Childhood Illness (IMCI) in 16 countries in Central Asia and Europe: implications for primary healthcare in the era of universal health coverage. Arch Dis Child 2019;104:1143–9. 10.1136/archdischild-2019-317072 PMC6900244 31558445

[22] Wynn JL , Polin RA. A neonatal sequential organ failure assessment score predicts mortality to late-onset sepsis in preterm very low birth weight infants. Pediatr Res 2020;88:85–90. 10.1038/s41390-019-0517-2 PMC7007331 31394566

[23] Sanchez-Pinto LN , Bennett TD, DeWitt PE et al; Society of Critical Care Medicine Pediatric Sepsis Definition Task Force. Development and validation of the phoenix criteria for pediatric sepsis and septic shock. Jama 2024;331:675–86. 10.1001/jama.2024.0196 PMC10900964 38245897

[24] NeoSep1: a study to determine the ranking of existing and new antibiotics combinations to treat newborn babies who are in hospital with severe sepsis 2022. ISRCTN Registry; ISRCTN48721236. https://www.isrctn.com/ISRCTN48721236 (14 April 2026, date last accessed).

[25] Hayes R , Hartnett J, Semova G et al; Infection, Inflammation, Immunology and Immunisation (I4) section of the European Society for Paediatric Research (ESPR). Neonatal sepsis definitions from randomised clinical trials. Pediatr Res 2023;93:1141–8. 10.1038/s41390-021-01749-3 PMC10132965 34743180

[26] Lutsar I , Trafojer UM, Heath PT et al; NeoMero Consortium. Meropenem vs standard of care for treatment of late onset sepsis in children of less than 90 days of age: study protocol for a randomised controlled trial. Trials 2011;12:215. 10.1186/1745-6215-12-215 PMC3193806 21958494

[27] Akdag A , Dilmen U, Haque K et al Role of pentoxifylline and/or IgM-enriched intravenous immunoglobulin in the management of neonatal sepsis. Am J Perinatol 2014;31:905–12. 10.1055/s-0033-1363771 24515621

[28] Mehta K , Bhatta NK, Majhi S et al Oral zinc supplementation for reducing mortality in probable neonatal sepsis: a double blind randomized placebo controlled trial. Indian Pediatr 2013;50:390–3. 10.1007/s13312-013-0120-2 23255688

[29] Banupriya N , Bhat BV, Benet BD et al Short term oral zinc supplementation among babies with neonatal sepsis for reducing mortality and improving outcome - a double-blind randomized controlled trial. Indian J Pediatr 2018;85:5–9. 10.1007/s12098-017-2444-8 28891027

[30] Dutta S , Nangia S, Jajoo M et al Comparison of efficacy of a 7-day versus a 14-day course of intravenous antibiotics in the treatment of uncomplicated neonatal bacterial sepsis: study protocol of a randomized controlled non-inferiority trial. Trials 2021;22:859. 10.1186/s13063-021-05785-6 PMC8628047 34844643

[31] Rohatgi S , Dewan P, Faridi MMA et al Seven versus 10 days antibiotic therapy for culture-proven neonatal sepsis: a randomised controlled trial. J Paediatr Child Health 2017;53:556–62. 10.1111/jpc.13518 28398692

