# Supplementary material for: Neonatal sepsis definitions for randomised–controlled trials: challenges and approaches
Source: Int J Epidemiol. 2026 Aug 18;55(5):dyag154. doi: 10.1093/ije/dyag154 (PMC13485345; doi:10.1093/ije/dyag154)
Supplement: Supplementary file 1 [file dyag154_supplementary_data.docx]

**Supplementary Materials**

1. Review Methods

Randomised controlled trials (RCTs) of interest, either protocols or full papers, were identified from a recently published systematic review (Hayes et al.; original systematic search conducted in May 2019, without date restrictions)(1), which evaluated trials that explicitly stated a definition for neonatal sepsis, either for eligibility or as an outcome measure.

Additional RCTs were identified by searching PubMed and ClinicalTrials.gov without date or language restrictions (last search conducted in September 2025; Medical Subject Heading (MeSH) and free-text terms for “infant, newborn” AND “neonatal sepsis” OR “newborn sepsis” OR “early-onset sepsis” OR “late-onset sepsis” OR “preterm infant sepsis” AND “randomised controlled trial” were combined), and through manual searches of reference lists.

One investigator (CM) extracted data, and two other reviewers (LFH, JAB) checked the extracted data.

2. Aim

We aimed to identify similarities and discrepancies within core domains in neonatal sepsis definitions used for trial eligibility, according to their sensitivity and specificity for detecting infants with neonatal sepsis.

3. Results

Twenty-nine RCTs providing a definition of neonatal sepsis as part of trial eligibility were reviewed(2-30).

We identified four core domains within the definition of neonatal sepsis: (i) clinical signs, (ii) laboratory markers, (iii) microbiological culture(s), and (iv) other (including requirement for antibiotic therapy and risk factors) (Table S1). There were ten combinations, with “culture positivity and/or suggestive clinical signs and/or laboratory markers” being the most represented (11/29, 37%).

Existing neonatal sepsis definitions, criteria or validated scores were explicitly referred to in six trials (21%). Study-specific definitions variably aligned with existing neonatal sepsis criteria. More than half of the considered RCTs (18/29, 62%) included microbiological results (Table S2), either positive or negative, in their sepsis definition for trial eligibility, with a consequent higher specificity.

The criterion of a positive microbiological culture for confirmed sepsis (15/29, 52%), also mentioned once as the sole criterion, was reported targeting either blood alone, most frequently, or other normally sterile body compartments (i.e. cerebrospinal fluid). The approach to coagulase-negative Staphylococci (CoNS) differed considerably CoNS were either excluded (8) or considered as pathogenic if isolated from two cultures from separate sites taken at the same time (12).

The remaining definitions targeted suspected sepsis with variable combinations of “clinical signs and/or laboratory markers and/or other” for trial enrolment and were therefore highly sensitive, but generally less specific (Tables S1-2). Among qualifying clinical signs, neurological signs (irritability, hypotonia, poor feeding, lethargy, seizures, etc.), respiratory signs (including apnoeic episodes, respiratory distress eventually requiring supplemental oxygen or increased respiratory support), gastrointestinal signs (feeding intolerance, abdominal distension, necrotising enterocolitis, etc.) and temperature instability were almost universally included. Moreover, trial definitions often encompassed cardiovascular signs (including an abnormal heart rate, hypotension or circulatory dysfunction/impaired peripheral perfusion) and general signs/miscellanea (bleeding, scleraema, purpura/petechiae, etc.). Three definitions additionally mentioned shock, and only one explicitly addressed organ dysfunction (14).

Even in low-resource settings (where provision was likely covered by the trial), laboratory investigations regularly encompassed raised inflammatory markers (acute phase reactants, mostly C-reactive protein), absolute neutrophil count (neutropenia), white blood cell count, immature/total neutrophil ratio, coagulation parameters such as platelet count (thrombocytopenia), glycaemic instability and metabolic acidosis, with cut-off values that were mostly aligned, although with some inter-study variability.

Other criteria included the requirement for antibiotics, maternal risk factors for early-onset sepsis (EOS) (positivity for Group B *Streptococcus*, fever, chorioamnionitis, premature and/or prolonged rupture of membranes, foul-smelling fluid), and neonatal risk factors for late-onset sepsis (LOS) (prematurity, low birth weight, prolonged mechanical ventilation, central venous catheter or parenteral nutrition, prolonged hospitalisation, use of steroids, prior exposure to broad spectrum antibiotics).

According to trial question, study design, setting, inclusion criteria and outcomes, 15 (52%) trials investigated efficacy, maximising internal validity and involving highly selected patient populations. Thirteen (45%) trials investigated effectiveness, with focus on generalisability and external validity in real-world conditions, reflecting routine clinical practice. There were 18 superiority and 10 non-inferiority trials. One trial investigated both effectiveness and efficacy for different outcomes (non-inferiority and superiority, respectively)(19).

Table S1. Combinations of core components/domains included in neonatal sepsis definitions in 29 randomised controlled trials.

|  | Clinical signs | Laboratory markers | Microbiological culture(s) | Other (antibiotic treatment, neonatal/maternal risk factors) | n. (%) |
| --- | --- | --- | --- | --- | --- |
| i) | ● | ● | ● |  | 11(37%) |
| ii) | ● | ● |  | ● | 5 (17%) |
| iii) | ● | ● |  |  | 3 (10%) |
| iv) | ● |  |  | ● | 2 (7%) |
| v) | ● | ● | ● | ● | 2 (7%) |
| vi) |  | ● | ● |  | 2 (7%) |
| vii) | ● |  | ● |  | 1 (3%) |
| viii) |  | ● |  | ● | 1 (3%) |
| ix) |  |  | ● |  | 1 (3%) |
| x) | ● |  | ● | ● | 1 (3%) |

Table S2. Characteristics of the 29 included trials and study-specific definitions of neonatal sepsis for trial eligibility or inclusion.

| Author  (Year of Publication) | | Haque, *et al*. (1988) (2) | Shenoi, *et al*. (1999) (3) | Bilgin, *et al*. (2001) (4) | Gitto, *et al*. (2001)(5) | Miura, *et al*. (2001) (6) | Chowdhary, *et al*. (2006) (7) | Lutsar, *et al*. (2011) (8) | Saini, *et al*. (2011) (9) | El Ganzoury, *et al*. (2012) (10) | Mehta, *et al*. (2013)(11) | Akdag, *et al*. (2014) (12) | Ceriani Cernadas, *et al.* (2014) (13) | Aradhya, *et al*. (2016) (14) | Banupriya, *et al*. (2016) (15) | Newton, *et al*. (2016) (16) | Rohatgi, *et al*. (2017) (17) | Shabaan, *et al*. (2017) (18) | Stocker, *et al*. (2017) (19) | Banupriya, *et al*. (2018) (20) | Baske, *et al*. (2018) (21) | Obiero *et al*. (2018) (22) | Saeidi, *et al*. (2019) (23) | Dutta, *et al*. (2021) (24) | Hill, *et al*. (2022) (25) | NeoSep1 (2022)(26) | Keij *et al*. (2022) (27) | Agudelo *et al.* (2023) (28) | Hidayah, *et al.* (2023) (29) | van der Weijden, *et al*. (2023) (30) | Total n. (%) |
| --- | --- | --- | --- | --- | --- | --- | --- | --- | --- | --- | --- | --- | --- | --- | --- | --- | --- | --- | --- | --- | --- | --- | --- | --- | --- | --- | --- | --- | --- | --- | --- |
| High-resource setting | | ● |  |  | ● | ● |  | ● |  |  |  |  | ● |  |  |  |  |  | ● |  |  |  | ● |  | ● |  | ● | ● | ● | ● | 12 (41%%) |
| Low-resource setting | |  | ● | ● |  |  | ● |  | ● | ● | ● | ● |  | ● | ● | ● | ● | ● |  | ● | ● | ● |  | ● |  |  |  |  |  |  | 16 (55%%) |
| Both high and low-resource setting | |  |  |  |  |  |  |  |  |  |  |  |  |  |  |  |  |  |  |  |  |  |  |  |  | ● |  |  |  |  | 1 (4%) |
| Existing sepsis definition/score | |  |  |  |  |  |  | ●  †; ‡ |  |  | ● §; ¶ |  |  |  |  |  |  | ●  \|\| |  |  |  |  |  |  | ●  ‡ | ●  * |  | ●  ֍ |  |  | 6 (21%) |
| Confirmed sepsis (as defined in individual studies) | | ● | ● | ● |  |  | ● | ● |  |  |  | ● | ● | ● | ● | ● | ● | ● |  |  | ● |  |  | ● | ● |  |  |  |  |  | 15 (52%) |
| Highly probable sepsis: blood culture + or - (as defined in individual studies) | |  |  |  | ● |  |  |  |  |  |  |  | ● |  |  |  |  |  |  |  |  |  |  |  |  |  |  |  |  |  | 2 (7%) |
| Suspected sepsis (as defined in individual studies) | | ● | ● |  | ● | ● |  | ● | ● | ● | ● | ● | ● |  | ● | ● |  |  | ● | ● | ● | ● | ● | ● | ● | ● | ● | ● | ● | ● | 24 (83%) |
| Clinical signs | Neurologic | ● | ● |  |  | ● | ● | ● | ● | ● |  | ● |  |  |  |  | ● |  | ● |  |  |  | ● |  | ● | ● | ● | ● |  | ● | 16 (55%) |
|  | Respiratory |  |  | ● | ● | ● |  | ● |  | ● |  | ● |  | ● |  |  | ● |  | ● |  |  |  | ● |  | ● | ● | ● |  | ● | ● | 15 (52%) |
|  | Cardiovascular |  |  |  | ● | ● |  | ● |  | ● |  | ● |  | ● |  |  |  |  | ● |  |  |  | ● |  | ● | ● | ● |  | ● | ● | 13 (43%) |
|  | Gastrointestinal | ● |  |  | ● | ● |  | ● |  | ● |  | ● | ● |  |  |  | ● |  | ● |  |  |  | ● |  | ● | ● | ● | ● | ● | ● | 16 (55%) |
|  | Temperature instability | ● |  | ● | ● | ● |  | ● |  | ● |  | ● | ● |  |  |  | ● |  | ● |  |  | ● | ● |  | ● | ● |  |  | ● | ● | 16 (55%) |
|  | Other signs  (general/miscellanea) | ● |  | ● |  | ● |  | ● |  | ● |  | ● | ● | ● |  | ● | ● |  |  | ● |  |  | ● | ● |  |  |  |  |  |  | 13 (45%) |
|  | Shock |  |  |  |  |  |  |  |  |  |  |  |  |  |  |  | ● |  |  |  |  |  |  |  |  | ● |  |  |  | ● | 3 (10%) |
|  | Organ dysfunction |  |  |  |  |  |  |  |  |  |  |  |  | ● |  |  |  |  |  |  |  |  |  |  |  |  |  |  |  |  | 1 (3%) |
| Laboratory markers | CRP or other acute phase reactant |  | ● |  | ● | ● |  | ● | ● |  | ● | ● | ● |  | ● | ● |  |  | ● | ● | ● |  |  |  | ● |  | ● | ● | ● |  | 17 (57%) |
|  | WBC count and/or absolute neutrophil count | ● | ● | ● | ● |  |  | ● |  | ● | ● | ● | ● | ● |  |  |  |  | ● |  | ● |  | ● |  | ● |  |  | ● | ● |  | 16 (55%) |
|  | Platelet count (thrombocytopenia) | ● | ● |  | ● | ● |  | ● |  |  |  |  | ● | ● |  |  |  |  |  |  |  |  |  |  | ● |  |  | ● | ● |  | 10 (35%) |
|  | I/T neutrophil ratio | ● |  |  |  |  |  | ● |  |  | ● |  | ● |  |  |  |  |  |  |  | ● |  | ● |  | ● |  |  | ● |  |  | 8 (28%) |
|  | Acidosis (base excess and/or lactate) |  |  |  |  |  |  | ● |  |  |  |  |  | ● |  |  | ● |  |  |  |  |  |  |  | ● |  |  |  |  |  | 4 (14%) |
|  | Glucose instability | ● |  |  |  |  |  | ● |  |  |  | ● |  |  |  |  | ● |  |  |  |  |  | ● |  | ● |  |  |  |  |  | 6 (21%) |
|  | Other laboratory markers |  |  |  |  | ● |  |  |  | ● |  |  |  | ● | ● | ● |  |  |  |  |  |  |  |  |  |  |  | ● |  |  | 6 (21%) |
| Microbiologic cultures | Blood | ● | ● | ● | ● | ● | ● | ● |  |  |  | ● | ● | ● | ● | ● | ● | ● |  |  | ● |  |  | ● | ● |  |  |  | ● |  | 18 (62%) |
|  | CSF | ● | ● |  |  |  |  | ● |  |  |  | ● | ● | ● |  |  |  | ● |  |  | ● |  |  |  |  |  |  |  |  |  | 8 (28%) |
|  | Urine |  | ● |  |  |  |  | ● |  |  |  |  |  |  |  |  |  | ● |  |  |  |  |  |  |  |  |  |  |  |  | 3 (10%) |
|  | Other sterile district |  |  |  |  |  |  | ● |  |  |  |  |  |  |  |  |  | ● |  |  |  |  |  |  |  |  |  |  |  |  | 2 (7%) |
| Other | Need for antibiotics |  |  |  |  | ● |  |  | ● |  |  |  |  |  |  |  |  |  | ● |  |  | ● |  | ● |  | ● | ● |  |  |  | 7 (24%) |
|  | Maternal/neonatal risk factors |  |  |  |  |  |  |  |  |  | ● |  |  |  |  |  |  |  | ● |  |  |  |  |  |  | ● | ● | ● |  | ● | 6 (21%) |
|  | Radiological evidence (i.e. pneumonia) |  |  |  |  |  |  |  |  |  | ● |  |  | ● |  |  |  |  |  |  |  |  |  |  |  |  |  |  |  |  | 2 (7%) |
| Superiority | | ● | ● | ● |  | ● |  | ● |  |  | ● | ● |  | ● | ● | ● |  | ● |  | ● | ● |  | ● |  |  | ● |  | ● | ● | ● | 18 |
| Non-inferiority | |  |  |  | ● |  | ● |  | ● | ● |  |  | ● |  |  |  | ● |  |  |  |  | ● |  | ● | ● |  | ● |  |  |  | 10 |
| Both superiority and non-inferiority | |  |  |  |  |  |  |  |  |  |  |  |  |  |  |  |  |  | ●  **  *** |  |  |  |  |  |  |  |  |  |  |  | 1 |
| Efficacy | |  |  |  | ● | ● |  | ● |  | ● |  |  |  | ● | ● | ● |  | ● |  | ● | ● |  | ● | ● | ● | ● | ● |  |  |  | 15 |
| Effectiveness | | ● | ● | ● |  |  | ● |  | ● |  | ● | ● | ● |  |  |  | ● |  |  |  |  | ● |  |  |  |  |  | ● | ● | ● | 13 |
| Both efficacy and effectiveness | |  |  |  |  |  |  |  |  |  |  |  |  |  |  |  |  |  | ●  **  *** |  |  |  |  |  |  |  |  |  |  |  | 1 |

Legend: High-resource setting: including high-income and upper-middle-income countries; Low-resource setting: including lower-middle-income and low-income countries;

†: IPSCC, International Paediatric Sepsis Consensus Conference;

‡: EMA, European Medicines Agency;

§: ICD, International Classification of Diseases;

¶: WHO, World Health Organisation;

||: CDC, Centers for Disease Control and Prevention;

֍: ASCON, Colombian Association of Neonatology; WBC, white blood count; CRP, C-reactive protein, I/Tneutrophil ratio, immature to total neutrophil ratio; CSF, cerebrospinal fluid;

*NeoSep severity score internally validated using a random subset of NeoObs data from 15% of babies that were not included in the development of the score.

** duration of antibiotic therapy *** reinfection of death in first month of life

4. References

1.	Hayes R, Hartnett J, Semova G, et al. Neonatal sepsis definitions from randomised clinical trials. *Pediatr Res* 2023;**93**(5):1141–8.

2.	Haque KN, Zaidi MH, Bahakim H. IgM-enriched intravenous immunoglobulin therapy in neonatal sepsis. *Am J Dis Child* 1988;**142**(12):1293–6.

3.	Shenoi A, Nagesh NK, Maiya PP, et al. Multicenter randomized placebo controlled trial of therapy with intravenous immunoglobulin in decreasing mortality due to neonatal sepsis. *Indian Pediatr* 1999;**36**(11):1113–8.

4.	Bilgin K, Yaramiş A, Haspolat K, et al. A randomized trial of granulocyte-macrophage colony-stimulating factor in neonates with sepsis and neutropenia. *Pediatrics* 2001;**107**(1):36–41.

5.	Gitto E, Karbownik M, Reiter RJ, et al. Effects of melatonin treatment in septic newborns. *Pediatr Res*. 2001;**50**(6):756–60.

6.	Miura E, Procianoy RS, Bittar C, et al. A randomized, double-masked, placebo-controlled trial of recombinant granulocyte colony-stimulating factor administration to preterm infants with the clinical diagnosis of early-onset sepsis. *Pediatrics* 2001;**107**(1):30–5.

7.	Chowdhary G, Dutta S, Narang A. Randomized controlled trial of 7-Day vs. 14-Day antibiotics for neonatal sepsis. *J Trop Pediatr* 2006;**52**(6):427–32.

8.	Lutsar I, Trafojer UM, Heath PT, et al. Meropenem vs standard of care for treatment of late onset sepsis in children of less than 90 days of age: study protocol for a randomised controlled trial. *Trials* 2011;**12**:215.

9.	Saini SS, Dutta S, Ray P, Narang A. Short course versus 7-day course of intravenous antibiotics for probable neonatal septicemia: a pilot, open-label, randomized controlled trial. *Indian Pediatr*. 2011;**48**(1):19–24.

10.	El-Ganzoury MM, El-Farrash RA, Saad AA, et al. In vivo effect of recombinant human granulocyte colony-stimulating factor on neutrophilic expression of CD11b in septic neonates: a randomized controlled trial. *Pediatr Hematol Oncol* 2012;**29**(3):272–84.

11.	Mehta K, Bhatta NK, Majhi S, et al. Oral zinc supplementation for reducing mortality in probable neonatal sepsis: a double blind randomized placebo controlled trial. *Indian Pediatr* 2013;**50**(4):390–3.

12.	Akdag A, Dilmen U, Haque K, et al. Role of pentoxifylline and/or IgM-enriched intravenous immunoglobulin in the management of neonatal sepsis. *Am J Perinatol* 2014;**31**(10):905–12.

13.	Ceriani Cernadas JM, Fernández Jonusas S, Márquez M, et al. Clinical outcome of neonates with nosocomial suspected sepsis treated with cefazolin or vancomycin: a non-inferiority, randomized, controlled trial. *Arch Argent Pediatr* 2014;**112**(4):308–14.

14.	Aradhya AS, Sundaram V, Kumar P, et al. Double Volume Exchange Transfusion in Severe Neonatal Sepsis. *Indian J Pediatr* 2016;**83**(2):107–13.

15.	Banupriya N, Vishnu Bhat B, Benet BD, et al. Efficacy of zinc supplementation on serum calprotectin, inflammatory cytokines and outcome in neonatal sepsis - a randomized controlled trial. *J Matern Fetal Neonatal Med* 2017;**30**(13):1627–31.

16.	Newton B, Bhat BV, Dhas BB, et al. Effect of Zinc Supplementation on Early Outcome of Neonatal Sepsis--A Randomized Controlled Trial. *Indian J Pediatr* 2016;**83**(4):289–93.

17.	Rohatgi S, Dewan P, Faridi MMA, et al. Seven versus 10 days antibiotic therapy for culture-proven neonatal sepsis: A randomised controlled trial. *J Paediatr Child Health* 2017;**53**(6):556–62.

18.	Shabaan AE, Nour I, Elsayed Eldegla H, et al. Conventional Versus Prolonged Infusion of Meropenem in Neonates With Gram-negative Late-onset Sepsis: A Randomized Controlled Trial. *Pediatr Infect Dis J* 2017;**36**(4):358–63.

19.	Stocker M, van Herk W, El Helou S, et al. Procalcitonin-guided decision making for duration of antibiotic therapy in neonates with suspected early-onset sepsis: a multicentre, randomised controlled trial (NeoPIns). *Lancet* 2017;**390**(10097):871–81.

20.	Banupriya N, Bhat BV, Benet BD, et al. Short Term Oral Zinc Supplementation among Babies with Neonatal Sepsis for Reducing Mortality and Improving Outcome - A Double-Blind Randomized Controlled Trial. *Indian J Pediatr* 2018;**85**(1):5–9.

21.	Baske K, Saini SS, Dutta S, Sundaram V. Epinephrine versus dopamine in neonatal septic shock: a double-blind randomized controlled trial. *Eur J Pediatr* 2018;**177**(9):1335–42.

22.	Obiero CW, Williams P, Murunga S, et al. Randomised controlled trial of fosfomycin in neonatal sepsis: pharmacokinetics and safety in relation to sodium overload. *Arch Dis Child* 2022;**107**(9):802–10.

23.	Saeidi R, Akhavan H, Tavakkolafshari J, Shapouri-Moghaddam A. Clinical and Biochemical Effects of Recombinant Human Granulocyte Colony-Stimulating Factor on the Prognosis of Preterm Infants with Early Onset Neonatal Sepsis. *Arch Pediatr Infect Dis* 2019;**7**(1):e67807.

24.	Dutta S, Nangia S, Jajoo M, et al. Comparison of efficacy of a 7-day versus a 14-day course of intravenous antibiotics in the treatment of uncomplicated neonatal bacterial sepsis: study protocol of a randomized controlled non-inferiority trial. *Trials* 2021;**22**(1):859.

25.	Hill LF, Clements MN, Turner MA, et al. Optimised versus standard dosing of vancomycin in infants with Gram-positive sepsis (NeoVanc): a multicentre, randomised, open-label, phase 2b, non-inferiority trial. *Lancet Child Adolesc Health* 2022;**6**(1):49–59.

26.	*NeoSep1: a study to determine the ranking of existing and new antibiotics combinations to treat newborn babies who are in hospital with severe sepsis 2022.* https://www.isrctn.com/ISRCTN48721236. (14 April 2026, date last accessed)

27.	Keij FM, Kornelisse RF, Hartwig NG, et al. Efficacy and safety of switching from intravenous to oral antibiotics (amoxicillin-clavulanic acid) versus a full course of intravenous antibiotics in neonates with probable bacterial infection (RAIN): a multicentre, randomised, open-label, non-inferiority trial. *Lancet Child Adolesc Health* 2022;**6**(11):799–809.

28.	*Effect of Kangaroo Care (Skin-to-skin Contact) on Heart Rate Variability in Preterm Infants With Late Neonatal Sepsis: a Randomized Crossover Clinical Trial. 2023.* https://clinicaltrials.gov/study/NCT05991648?term=NCT05991648%20&rank=1 (14 April 2026, date last accessed)

29.	*The Effect of Melatonin as an Adjuvant Therapy for Preterm Neonates With Sepsis. 2023.* https://clinicaltrials.gov/study/NCT06191523?term=NCT06191523&rank=1 (14 April 2026, date last accessed)

30.	van der Weijden BM, van der Weide MC, Plötz FB, Achten NB. Evaluating safety and effectiveness of the early-onset sepsis calculator to reduce antibiotic exposure in Dutch at-risk newborns: a protocol for a cluster randomised controlled trial. *BMJ Open*. 2023;**13**(2):e069253.
